# Two-decade change in prevalence of cognitive impairment in the UK

**DOI:** 10.1007/s10654-019-00554-x

**Published:** 2019-09-05

**Authors:** Connor Richardson, Blossom C. M. Stephan, Louise Robinson, Carol Brayne, Fiona E. Matthews, Cognitive Function and Ageing Study Collaboration

**Affiliations:** 1 Newcastle University Institute for Ageing and Institute for Health and Society, Newcastle University, Newcastle Biomedical Research Building, Newcastle upon Tyne, NE4 5PL UK; 2 Division of Psychiatry and Applied Psychology, Institute of Mental Health, School of Medicine, University of Nottingham, Innovation Park, Nottingham, NG7 2TU UK; 3 Cambridge Institute of Public Health, University of Cambridge School of Clinical Medicine, Forvie Site, Cambridge Biomedical Campus, Cambridge, CB2 0SR UK

**Keywords:** Mild cognitive impairment, Cognition, Dementia, Alzheimer’s disease, Epidemiology, MCI

## Abstract

Identification of individuals at high risk of dementia has usually focused attention on the clinical concept of mild cognitive impairment (MCI), which captures an intermediate state between normal cognitive ageing and dementia. In many countries age specific risk of dementia has declined, but whether this is also the case for subclinical cognitive impairment is unknown. This has important implications for prevention, planning and policy. Here we describe subclinical cognitive impairment and mild dementia prevalence changes, in the UK, over 2 decades. The Cognitive Function and Ageing Studies have examined the full spectrum of cognition, from normal to dementia, in representative populations of people aged ≥ 65 years in the UK over the last 2 decades 7635 participants were interviewed in CFAS I in Cambridgeshire, Newcastle, and Nottingham in 1991, with 1457 being diagnostically assessed. In the same geographical areas, the CFAS II investigators interviewed 7796 individuals in 2011. Using established criteria, the population was categorised into seven groups: no cognitive impairment, Mild cognitive Impairment (defined using consensus criteria), other cognitive impairment no dementia without functional impairment, OCIND with functional impairment, cognitive impairment (MMSE < 24 and no functional impairment), mild dementia (MMSE < 24 with functional impairment, not captured by CFAS dementia criteria), and CFAS dementia criteria. Multinomial logistic regression, adjusted for age and sex, was used to estimate the prevalence of impairment in both studies. Results were standardized to the age-sex specific UK and global population. There is a clear increase in the prevalence of other cognitive Impairment no Dementia (without functional impairment), with the purer MCI remaining stable. In the UK, mild dementia is estimated to fall from 520,704 cases (5.7%, 95% CI 3.8, 8.1) in 1991 to 315,142 (3.0%, 95% CI 2.4, 3.8) in 2011, cognitive impairment, has fallen from 1,225,984 (13.5%, 95% CI 10.1, 17.5) to 654,436 (6.3%, 95% CI 5.4, 7.3) cases. Using additional categories which reflect the continuum of cognitive decline and impairment in populations we see that the mildest dementia declines, but that there is stability in estimates of those who meet MCI criteria. Increases were found in the Other Cognitive Impairment no Dementia group. The decline observed in severe impairment thus seems to have resulted in larger proportions of the population in milder forms, seen alongside physical illnesses.

**Electronic supplementary material:**

The online version of this article (10.1007/s10654-019-00554-x) contains supplementary material, which is available to authorized users.

## Introduction

Mild cognitive impairment (MCI) represents an intermediate state of cognitive functioning between changes expected as a normal part of ageing and dementia [1]. MCI prevalence has been estimated to range between < 1 and 42% in older populations depending on the classification criteria used and setting (e.g. clinical vs. population based) [2–4]. Reported rates of progression of MCI to dementia have also varied and range from around 8–15% per year [2]. However, not all individuals with MCI experience decline; MCI has been shown to be a transient state, with some of the MCI population at risk of progressing to dementia at a later stage while others possibly regain cognitive function [5–9].

Defining subclinical cognitive impairment is complex and challenging due to a lack of standardised diagnostic criteria. Although the MCI criteria developed by Petersen and colleagues are an attempt to standardise subclinical impairment, concerns have emerged that the criteria are too restrictive [10, 11]. Population based studies have repeatedly shown that definitions of cognitive decline which cover a broader range of dysfunction, such as other cognitive impairment no dementia (OCIND) are more prevalent than more restrictive definitions of MCI and also have a high progression rate to dementia [6, 12]. There is also disagreement about the cut points used to define deficits in cognitive performance needed to distinguish MCI from minimal or questionable dementia [13, 14]. The longstanding approach of excluding individuals with medical comorbidities has also been challenged, with evidence suggesting that excluding this group could result in insufficient or biased MCI diagnosis, especially for clinical trials, thus restricting generalizability of results [15]. Consensus criteria proposed by the International Working Group on Mild Cognitive Impairment, in 2004, broadened the concept developed by Petersen and colleagues to include impairment in any cognitive domain as well as relax criteria focused on functional impairment [3]. In 2011, the National Institute on Aging and the Alzheimer’s Association (NIA-AA) refined the definition of MCI to account for possible aetiologies, including amnestic MCI (aMCI) and non-amnestic MCI (naMCI) thought to be associated with AD and vascular dementia, respectively [16]. In 2013 the new Diagnostic and Statistical Manual of Mental Disorders, Fifth Edition (DSM-V) was released and included the diagnosis of mild neurocognitive disorder, a pre-dementia state based on MCI criteria [17]. Few community based populations have investigated DSM-V mild neurocognitive disorder, however a prevalence of 20% (95% CI 17.8–23.0) was reported in the LIFE-Adult-Study [18]. This study also found a 98.6% diagnostic overlap between the DSM-V and consensus MCI criteria.

Cohort effects have been observed in dementia. Indeed, in high income countries such as USA and UK, in the last 2 decades, it appears that risk of dementia is declining [19]. Whether cohort effects are seen in the prevalence of MCI and risk of progression to dementia is not known but has important public health implications (e.g. calculating population burden of disease). Further, determining how best to identify individuals at high risk of dementia will have important implications for intervention trials (e.g. clinical trial recruitment protocols), treatment (e.g. personalised medicine), public health surveillance (e.g. monitoring) and education (e.g. prevention programmes). Therefore, in this study, MCI (defined using consensus criteria) [20], in addition to other states of the entire cognitive spectrum, are investigated in the Cognitive Function and Ageing Studies (CFAS) in order to determine whether similar trends are seen for these states in the last 2 decades as those seen in dementia.

## Methods

### Setting, study design, and participants

**Fig. 1:**
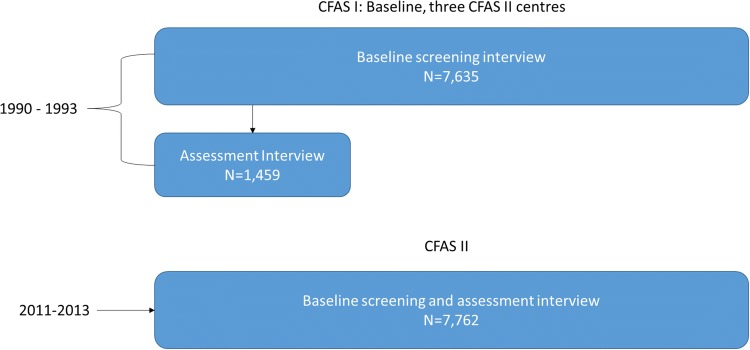
CFAS I and CFAS II study design

Information on the CFAS I and II study designs has been published in detail previously [21, 22]. In brief, CFAS I baseline interviews were undertaken between 1989 and 1994. In total, 13,044 individuals aged 65 years and older were recruited in five geographical areas in England and Wales including: Cambridgeshire, Oxfordshire, Nottingham, Newcastle and Gwynedd. Between 2008 and 2011 a new cohort of individuals (N = 7796), aged 65 years and older were selected and interviewed from three of the original CFAS areas: Cambridgeshire, Newcastle, and Nottingham with the aim to provide contemporary evidence on dementia prevalence, incidence and risk factors. This study of prevalence includes all participants who underwent diagnostic assessment in CFAS I and CFAS II (Fig. 1).

### Ethics

Fully informed written consent was sought, and when capacity was impaired procedures complied with the UK Mental Capacity Act 2005. Full details of ethical approvals in place for CFAS I and II can be found at: http://www.cfas.ac.uk/cfas-i/data/#cfasi-ethical-approval.

### Outcome description

**Fig. 2:**
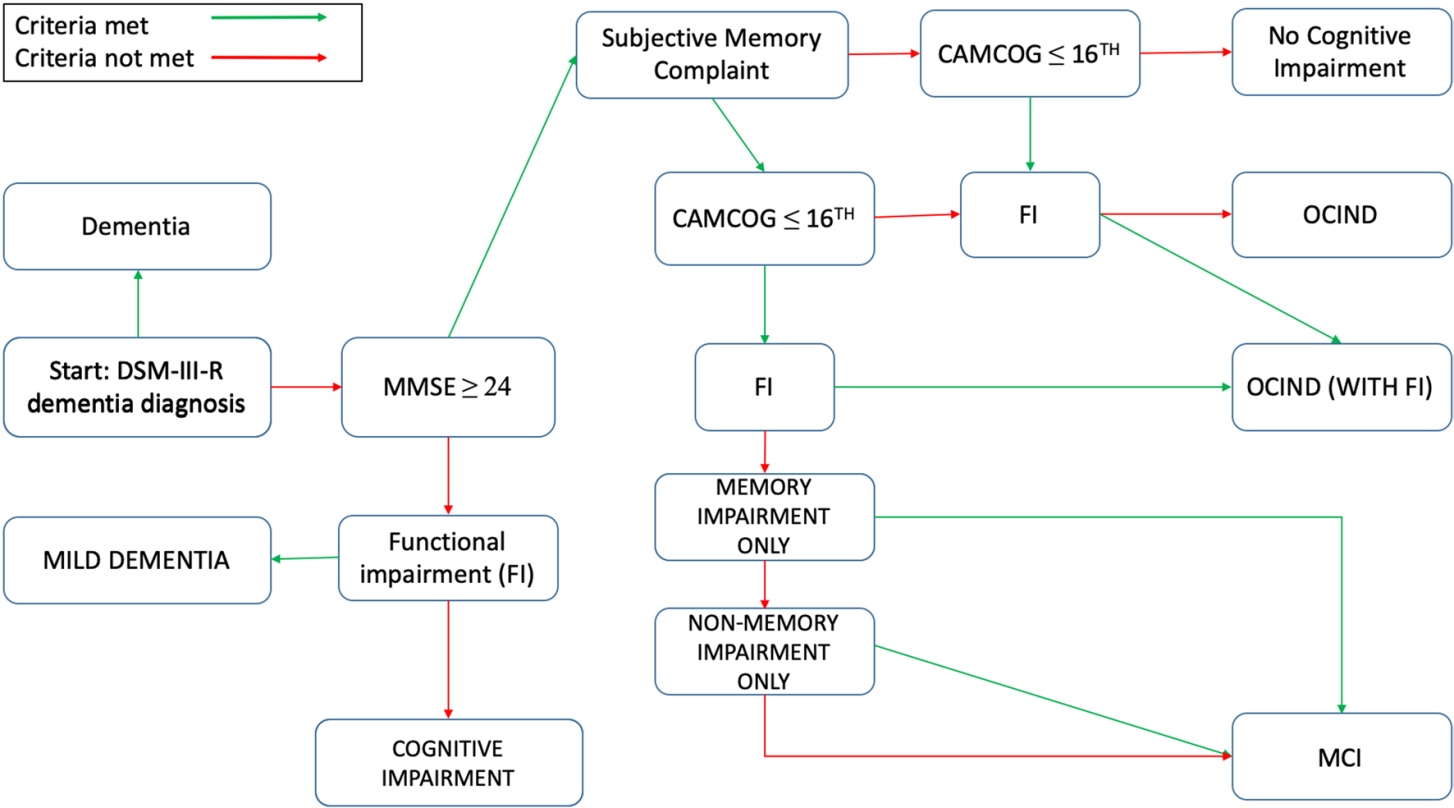
Flow chart describing diagnostic criteria for definitions of cognitive spectrum. Dementia diagnosed using GMS AGECAT diagnosis—DSM-III-R, subjective memory complaint was measured through informant or proxy interview for evidence of memory impairment or decline over 6 months. Global cognitive impairment was measured using the Mini Mental State Examination (MMSE) with impairment established by scoring below 24, domain specific impairment was measured using the CAMCOG examination, and impairment was defined as ≤ 16th percentile using education adjusted percentile regression modelling. Disability (FI) was defined as having impairments in BADL’s. Amnestic, non-amnestic and multiple domain MCI was established using memory and non-memory components of the CAMCOG, for this analysis Revised MCI criteria was defined by having aMCI, naMCI or mMCI

Full details of the cognitive outcomes are described in the Appendix. Briefly we sought to classify the entire cognitive spectrum of the older population from normal cognition, through mild cognitive impairment to dementia. Those without study diagnosis of dementia were classified into groups: No cognitive impairment, MCI, other cognitive impairment not dementia (OCIND—with and without functional impairment) and cognitive impairment with (mild dementia) and without functional impairment (Supplementary Table 1, Fig. 2).

### Statistical analysis

Prevalence estimates were obtained using a multinomial logistic regression model, which predicted the probability of participants belonging to each group within the cognitive spectrum in one model. Participants with no cognitive impairment were treated as the reference group. This was repeated for men and women in each 5-year age band. Predicted probabilities were multiplied by UK population, stratified by age and sex to estimate population prevalence [22]. Inverse probability weights were used to adjust for non-response (CFAS I and CFAS II) and screen-assessment sampling (CFAS I), based on UK age, sex and deprivation structure. The three centres (Cambridgeshire, Newcastle and Nottingham) included in both CFAS I (n = 7635) and II (n = 7796) were used. Bootstrap resampling with 1000 replications was used to calculate 95% confidence intervals (95% CI) via the [bias corrected and accelerated] percentile method.

Age and sex specific prevalence estimates were combined with population data to obtain the number of individuals in the population in each cognitive group in 1991 (using CFAS I estimates) and in 2011 (using CFAS II estimates) [23]. Predicted estimates of the population assuming a constant prevalence over the 2 decades was calculated using the CFAS I estimates combined with the 2011 UK population data.

## Results

### Sample demographics

In CFAS I 7635 individuals took part in the baseline wave (80% response), with 1459 taking part in the wave 1 assessment interview (82% of those still alive), giving an overall response rate of 66% to the assessment interview. In CFAS II screen and assessment was undertaken in one stage and had a response rate of 56%. Figure 1 shows the number of individuals included in the baseline interviews in CFAS I and II. Demographic characteristics including age, sex, location, education, baseline cognitive function, Basic Activities of Daily Living/Instrumental Activities of Daily Living and dementia status are shown in Table 1.

**Table 1:** Baseline sociodemographic characteristics of CFAS I and II

	CFAS I		CFAS II
	Screening (%)^a^	Assessment (%)^b^	N (%)^c^
*Sex*			
Men	3045 (39)	531 (38)	3550 (44)
Women	4590 (61)	926 (62)	4246 (56)
*Age group*			
65–69	1981 (25)	310 (23)	1939 (23)
70–74	1776 (23)	320 (22)	1874 (23)
75–79	1725 (22)	263 (23)	1623 (21)
80–84	1308 (18)	291 (20)	1289 (17)
85–89	615 (9)	186 (9)	769 (11)
> 90	230 (4)	87 (3)	302 (6)
*Education (years full time)*			
0–9	5529 (74)	1074 (80)	1976 (26)
10	644 (9)	106 (8)	2734 (36)
> 10	1286 (17)	172 (12)	2909 (38)
*Geographical area*			
Cambridgeshire	2601 (34)	465 (37)	2558 (30)
Newcastle	2522 (33)	499 (31)	2616 (34)
Nottingham	2512 (33)	493 (32)	2622 (35)
*Residential status*			
Community	7281 (95)	1269 (95)	7599 (97)
Care homes	347 (5)	183 (5)	197 (3)
Dementia	NA	329 (8.6)	461 (6)

Characteristics (n (%)) including sex, age group, education (years full time), geographical area, residential status and dementia status. Percentages are backed weighted for initial non-response (CFAS I screening and CFAS II) and study design (CFAS I assessment)

Data are n (%)

CFAS, Cognitive Function and Ageing Study

^a^Percentages back-weighted for non-response

^b^Percentages back-weighted for sampling design and non-response

^c^Residential status missing for seven individuals for CFAS I (of whom five were also assessed)

### UK population prevalence of each cognitive group

**Fig. 3:**
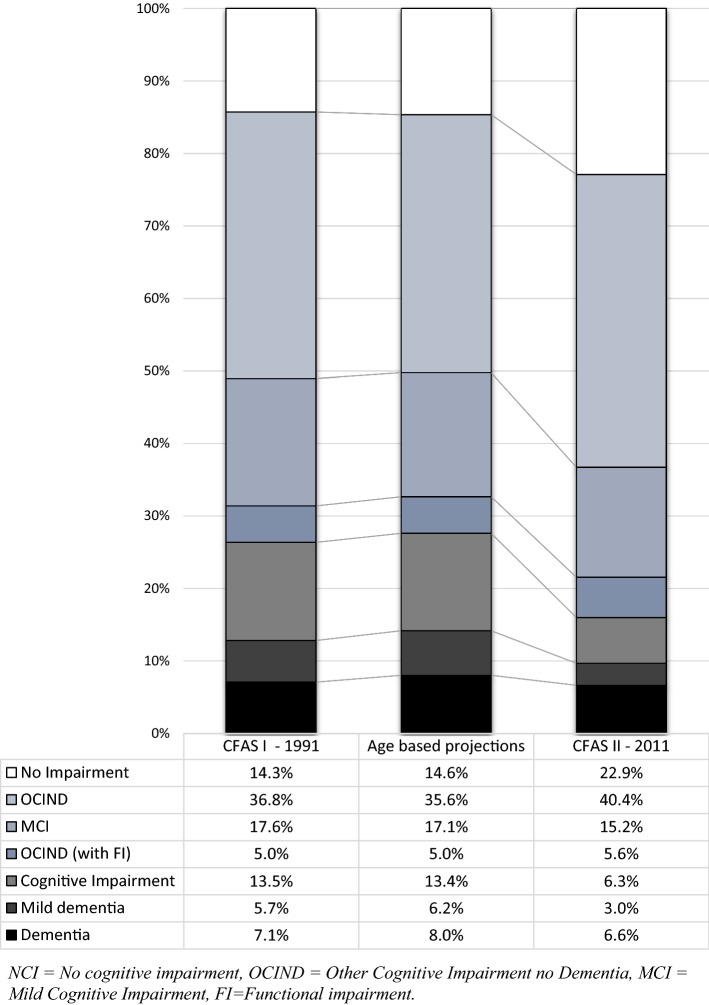
Prevalence of cognitive impairment standardized to UK population age and sex structure (%). Estimates were obtained using multiple regression modelling, age based projections for 2011 were obtained using 1991 estimates applied to 2011 population. NCI, no cognitive impairment, OCIND, other cognitive impairment no dementia, MCI, mild cognitive impairment, FI, functional impairment

Figure 3 shows the prevalence of each cognitive group standardized to the age and sex structure of the UK in 1991 and 2011. The figure also shows the estimated prevalence for 2011 from age and sex-based predictions from the 1991 data. Cohort effects were observed in the definitions of mild dementia and cognitive impairment. Over the 2 decades, mild dementia has almost halved from 520,704 cases (5.7%, 95% CI 3.8, 8.1) in 1991 to 315,142 cases (3.0%, 95% CI 2.4, 3.8) in 2011 (Table 2). There has been a larger decrease in cognitive impairment, which has proportionally fallen by more than half from 1,225,984 (13.5%, 95% CI 10.1, 17.5) to 654,436 cases (6.3%, 95% CI 5.4, 7.3) (Table 2). This contrasts with the age-based projections which predicted that the prevalence of mild dementia and cognitive impairment would have remained relatively stable at 6.2% (95% CI 4.1, 8.7) and 13.4% (95% CI 9.9, 17.5), respectively (Table 2). The reduction in MCI prevalence over the 2 decades was small (2.4%) suggesting stable case numbers: 1,590,481 cases (17.6%, 95% CI 12.5, 22.9) versus 1,575,577 cases (15.2%, 95% CI 13.8, 16.6). In contrast between 1991 and 2011, OCIND (without FI) prevalence increased from 3,331,809 (36.8%, 95% CI 30.3, 43.6) to 4,191,265 cases (40.4%, 95% CI 38.5, 42.3) while prevalence of OCIND (with FI) remained stable (5.0%, 95% CI 2.6, 8.1 in 1991 vs. 5.6%, 95% CI 4.6, 6.5 in 2011) (Table 2). The numbers of people with no cognitive impairment increased from 1,291,396 cases (14.3%, 95% CI 9.3, 19.4) in 1991, to an estimated 2,376,070 cases (22.9%, 95% CI 21.3, 24.5), this result was also higher than the predicted age-based projections for 2011 of 1,518,700 cases (14.6%, 95% CI 9.6, 19.9).

**Table 2:** Prevalence of cognitive impairment standardized to UK population age and sex structure

Cognitive group	CFAS I		2011 Age based projections		CFAS II	
	n	% (95% CI)	n	% (95% CI)	n	% (95% CI)
NCI	1,291,396	14.3 (9.3, 19.4)	1,518,700	14.6 (9.6, 19.9)	2,376,070	22.9 (21.3, 24.5)
OCIND	3,331,809	36.8 (30.3, 43.6)	3,695,737	35.6 (29.2, 42.5)	4,191,265	40.4 (38.5, 42.3)
MCI	1,590,481	17.6 (12.5, 22.9)	1,775,397	17.1 (12.1, 22.4)	1,575,577	15.2 (13.5, 16.6)
OCIND (with FI)	456,253	5.0 (2.6, 8.1)	522,149	5.0 (2.6, 8.1)	577,559	5.6 (4.6, 6.5)
Cognitive impairment	1,225,984	13.5 (10.1, 17.5)	1,395,954	13.4 (9.9, 17.5)	654,436	6.3 (5.4, 7.3)
Mild dementia	520,704	5.7 (3.8, 8.1)	638,522	6.2 (4.1, 8.7)	315,142	3.0 (2.4, 3.8)
Dementia	642,238	7.1 (5.3, 9.2)	833,426	8.0 (6.1, 10.4)	690,051	6.6 (5.6, 7.8)

Estimates were obtained using multiple regression modelling, with 95% CI’s obtained by bootstrap with 1000 replications via the percentile method. Age based projections for 2011 were obtained using 1991 estimates applied to 2011 population

## Discussion

Using CFAS I and II the prevalence of the full spectrum of cognitive function including normal, MCI, OCIND and dementia (including its mildest forms) could be measured over 2 decades with population resampling. There is clear evidence of a cohort effect in the prevalence of mild dementia (decrease), cognitive impairment (decrease) and OCIND without functional impairment (increased). The numbers of people with no impairment have also increased relative to the age-based projections for 2011. Cohort effects are not seen in MCI and OCIND (with functional impairment), the prevalence of which appear to remain stable over the 2 decades.

There are a number of strengths to the study. This study is able to investigate cognitive impairment across the full spectrum (i.e. from no impairment to dementia), strengthened by the methodological approach of using repeated prevalence’s studies. CFAS give unique insight based on random sampling from three areas of England, allowing the examination of cross-generational cognitive impairment, being powered to detect changes and implementation of consistent methods and diagnostic procedures. There are also some limitations. First, although the key methodologies of CFAS I and II are identical, CFAS I comprised a two-stage design with the complete cognitive assessment on a subsample, though the analysis does take this fully into account. CFAS II was designed to avoid this complexity while retaining the ability to compare results across the two studies. Second, dementia was diagnosed algorithmically using the AGECAT validated with DSM-III-R. With the introduction of new DSM-V criteria, it is unknown whether prevalence of dementia or MCI may have differed had these criteria been applied. Understanding population change in MCI has been complicated by the lack of standardised cut-off scores, age norms, changing criteria including DSM-V creating an unstable environment. The advantage of using population based data is that it was collected without applying criteria and can be used to operationalise criteria that were not developed at the time of the study. Here we used consensus criteria for MCI which has been shown that when compared with the recent DSM-V criteria of mild neurocognitive disorder has high diagnostic overlap (98.6%) [17, 20].

While cohort effects were observed across the spectrum of cognitive impairment there were differences depending on group. Cognitive impairment and mild dementia do not share the same level of decrease as has been previously observed in dementia with both groups seeing a far greater reduction. When we combine the estimates of dementia, mild dementia and cognitive impairment in the older population together it corresponds to over one million people in 2018 with many likely to have very similar care needs to those with a diagnosis of dementia. In contrast, the proportion of cases with no cognitive impairment and OCIND (without FI) increased. A possible explanation for this is that the trends in population ageing along with improvements in education, health care and reduction of cardiovascular risk factors associated with cognitive impairment have led to a greater proportion of older people with no impairment. Further, where cognition has declined it remains mild rather than declining to the point of impairment. Interestingly the prevalence of MCI has remained stable since 1991. The estimated prevalence is consistent with other large cohort studies internationally, with the average prevalence of MCI from consensus criteria around 12% and 18% [24–28].

There is much debate in the literature about the variance in MCI prevalence estimates from epidemiological studies [2]. Evidence suggests that lack of consistency is largely due to a lack of standardised diagnostic criteria and homogenous study designs, methodology and populations [20]. For decades, the main area of disagreement has been around how to define mild impairment by neuropsychological testing; for example, tighter definitions such as scoring 1.5 standard deviations from normal on memory domain tests show much lower prevalence’s compared to the DSM-V criteria of between 1 and 2 standard deviations on non-specified domain testing [4]. Recently the criteria for MCI has narrowed with the introduction of consensus criteria [20] and different subtypes (amnestic MCI, non-amnestic MCI and multiple MCI) with the aim to help improve diagnostic accuracy [2]. Consensus criteria were selected for this study, for reasons stated earlier, and different changes over time may be obtained if a different definition were used. But this is beyond the scope of the current study.

The results show that since 1991 there has been a shift in the cognitive burden within the older population; although the prevalence of dementia and its mild dementia forms may be declining, OCIND is increasing and MCI is stable. These results have a number of implications for strategic planning for cognitive impairment and dementia. First, they provide further evidence alongside studies [29–31] that have found a decline in risk of cognitive impairment (measured using CIND) in older aged populations; extending the findings for the first time by showing that although severe cognitive impairment and the mildest forms of dementia have decreased, mild impairment in the form of OCIND has increased and MCI has remained stable. Strategic planning for the numbers of people with dementia will have to be adjusted to budget correctly as the prevalence of the disease declines, however, this analysis shows that despite these trends, there is still a significant number of people with cognitive impairment and mild dementia who may indeed be missed from formal dementia diagnosis but who have similar care needs. Planning for increasing numbers of people with OCIND and MCI is more complex, evidence suggests patients diagnosed with MCI prefer to undergo testing to predict dementia prevalence in the future; due to ever changing technologies, guidelines for diagnosis and management for MCI means testing is likely to be uncertain [32]. Diagnostic testing for MCI can incur heavy financial costs, and so far interventions to prevent or delay progression to dementia have limited efficacy [33, 34]. Planning for the future must involve policy makers and clinicians discussing the balance of risks and benefits of interventions, also involving patient’s views on interventions in later life.

In addition, our results suggest that changes seen in the population that has prevented dementia, has also impacted on severe cognitive impairment. However, what is not clear is whether lifestyle factors such as education and improved cardiovascular and metabolic health is having the same reductive effect on MCI [30, 35]. Indeed, the findings suggest that the increase in OCIND and stability of MCI could reflect “normal cognitive ageing” among a population which is substantially older. As well as the financial burden, a diagnosis of MCI can lead to emotional distress and social stigma; cognitive decline is a subject of fear among older people and patients may thus prefer foregoing the diagnosis altogether [33, 36]. In light of this care must be taken not to wrongly identify people with MCI as a clinical condition, which may be “normal cognitive ageing”. Clinical decision making regarding MCI will likely change in light of an effective disease modifying agent for pre-dementia being identified. In the meantime evidence suggests it is through healthy lifestyle behaviours, better educational attainment, minimizing risks from polypharmacy and comorbidities in the wider population which should be further encouraged to reduce risk of severe cognitive impairment [30].

## Electronic supplementary material

Below is the link to the electronic supplementary material.

Supplementary material 1 (DOCX 154 kb)
