# Supplementary material for: Two-decade change in prevalence of cognitive impairment in the UK
Source: Eur J Epidemiol. 2019 Sep 5;34(11):1085–92. doi: 10.1007/s10654-019-00554-x (PMC6861197; doi:10.1007/s10654-019-00554-x)
Supplement: Supplementary file 1 — Supplementary material 1 (DOCX 154 kb) [file 10654_2019_554_MOESM1_ESM.docx]

**Supplementary appendix**

**Setting, study design, and participants**

In both studies, the population for invitation to interview were randomly sampled from primary care registers in each geographical area. Sampling was stratified according to age group (65–74 years’ vs ≥75 years; 1,250 per stratum per area). Oversampling was used to allow for death, incorrect registration, ineligibility, general practitioner refusals, and participant or gatekeeper refusal. The primary care practices screened records of patients in selected samples regularly for deaths and terminal illness. An introductory letter from the general practitioner was followed by a visit by a named study interviewer.

**Outcome definitions**

CFAS I & II participant’s cognitive and functional diagnostic criteria are illustrated in figure 1.

**Dementia**

Dementia status was assessed using the Geriatric Mental State (GMS) AGECAT (Automated Geriatric Examination for Computer Assisted Taxonomy) algorithm. This gives a similar diagnosis as DSM-III-R criteria. [^1^](#_ENREF_1)

**MCI definition**

MCI was operationalized using consensus criteria [^2^](#_ENREF_2) which includes: subjective memory complaint (SMC), normal global cognitive function, impaired domain specific cognitive function and essentially normal physical functioning. These criteria were implemented as previous studies have found that they have good predictive accuracy for dementia in population-based settings. In addition, they mirror the core DSM-V and NIA-AA criteria, with the exception being that biomarkers proposed for mapping the later criteria are not available in CFAS [^3^](#_ENREF_3) (table 1).

SMC was regarded as present if participants, or their informant, reported memory problems: a combined score was created from three questions including: (1) Have you had any difficulty with your memory? (2) Have you tended to forget things recently? And, (3) has he or she had any difficulty with his or her memory? Answers for each question were coded as yes or no, from which individuals were dichotomized into noncomplainers or complainers (positive response to one or more questions). Normal global cognitive function was defined as a MMSE score ≥24 [^4^](#_ENREF_4). Domain specific cognitive function, including memory and non-memory performance, was assessed using the Cambridge Cognitive Examination (CAMCOG). Test scores on the memory (i.e. learning, recent and remote) and non-memory (i.e. orientation, language, attention/ calculation, praxis, abstract thinking, or perception) sub-scales were combined and impairment defined as a score ≤ 16^th^ percentile (i.e. approximately one standard deviation [SD] below the mean), adjusting for age and education using quantile regression analysis. This is equivalent to one standard deviation for non-normally distributed data. This cut off was chosen as a previous CFAS study found that when compared to 1 SD the 1.5 SD cut off had the effect of reducing sensitivity without significantly increasing specificity [^4^](#_ENREF_4).

**Functional impairment**

Activities of daily living (basic (BADL) and instrumental (IADL)), were measured using questions from the Modified Townsend Disability Scale, with an additional three items [^5^](#_ENREF_5). Using information on a hierarchy of activities of daily living/instrumental activities of daily living (BADL/IADL) disability, individuals were classified into one of three groups. The first group included those individuals who showed no evidence of impairment in BADL or IADLs on items including washing, cooking hot meals, putting on shoes and socks, completing heavy housework or shopping, and carrying heavy bags, and the individual can get around outside. The second group included individuals with impairments only in IADLs, including those individuals who require regular help on items including heavy housework or shopping and carrying heavy bags. The third group included those individuals with deficits in basic activities of daily living including individuals who require help at least several times per week on items relating to washing, cooking, and dressing or if they are house/chair bound [^5^](#_ENREF_5). Good physical functioning, required for a diagnosis of MCI, was defined as not needing assistance with basic ADL’s only.

**NCI Group**

To be classified as NCI, individuals had to have: a MMSE score ≥24, all CAMCOG domains >16^th^ centile, and no dementia.

**OCIND (with and without Functional Impairment)**

To be classified as OCIND (without FI) individuals had to have an MMSE score ≥24, any CAMCOG domains ≤ 16^th^ centile, no functional impairment and no dementia.

Individuals who met the cognitive components but did have functional impairments were classified as OCIND (with FI).

**Cognitive impairment and mild dementia groups**

Individuals were classified as having cognitive impairment if they had a MMSE < 24 and no functional impairments. For the purposes of this paper the cognitive impairment definition includes those who otherwise may be classified as a moderate or severe cognitive impairment. If participants had both moderate cognitive impairment and functional impairments they were classified into the mild dementia category.

***Figure 1: Flow chart describing diagnostic criteria for definitions of cognitive spectrum.***


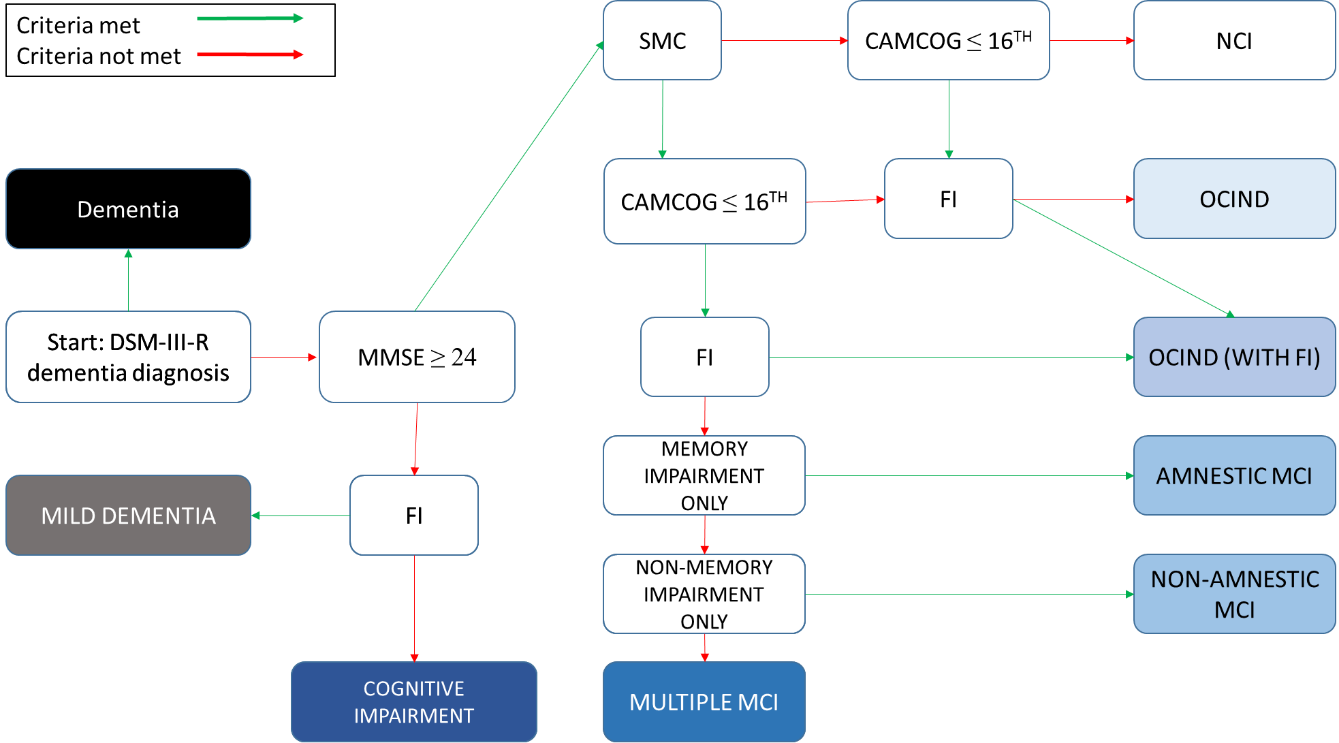


*Dementia diagnosed using GMS AGECAT diagnosis - DSM-III-R, subjective memory complaint was measured through informant or proxy interview for evidence of memory impairment or decline over 6 months. Global cognitive impairment was measured using the Mini Mental State Examination (MMSE) with impairment established by scoring below 24, domain specific impairment was measured using the CAMCOG examination, and impairment was defined as ≤ 16^th^ percentile using education adjusted percentile regression modelling. Disability (FI) was defined as having impairments in BADL’s. amnestic, non-amnestic and multiple domain MCI was established using memory and non-memory components of the CAMCOG, for this analysis Revised MCI criteria was defined by having aMCI, naMCI or mMCI.*

***Table 1: Core diagnostic criteria for MCI diagnosis***

| Definitions | Original mayo clinic | Expanded/ Consensus criteria | NIA-AA | DSM-V |
| --- | --- | --- | --- | --- |
| Criteria |  |  |  |  |
| Self or informant reported memory complaint | **X** |  |  |  |
| Self or informant reported cognitive complaint |  | **X** | **X** | **X** |
| Objective memory Impairment | **X** |  |  |  |
| Objective cognitive impairment |  | **X** | **X** | **X** |
| Essentially preserved general cognitive functioning | **X** |  |  |  |
| Preserved independence in functional abilities | **X** | **X** | **X** | **X** |
| No dementia | **X** | **X** | **X** | **X** |

*NIA-AA= National Institute for Ageing-Alzheimer's Association, DSM= Diagnostic and Statistical Manual*

1. Copeland JR, Dewey ME, Griffiths-Jones HM. A computerized psychiatric diagnostic system and case nomenclature for elderly subjects: GMS and AGECAT. Psychol Med 1986;16:89-99.

2. Winblad B, Palmer K, Kivipelto M, et al. Mild cognitive impairment--beyond controversies, towards a consensus: report of the International Working Group on Mild Cognitive Impairment. J Intern Med 2004;256:240-6.

3. Petersen RC, Caracciolo B, Brayne C, Gauthier S, Jelic V, Fratiglioni L. Mild cognitive impairment: a concept in evolution. Journal of Internal Medicine 2014;275:214-28.

4. Stephan BCM, Savva GM, Brayne C, Bond J, McKeith IG, Matthews FE. Optimizing Mild Cognitive Impairment for Discriminating Dementia Risk in the General Older Population. The American Journal of Geriatric Psychiatry 2010;18:662-73.

5. McGee MA, Johnson AL, Kay DWK, subcommittee MCA. The description of activities of daily living in five centres in England and Wales: THE MEDICAL RESEARCH COUNCIL COGNITIVE FUNCTION AND AGEING STUDY (MRC CFAS). Age and Ageing 1998;27:605-13.
